# ‘I guess I’ll wait to hear’—communication of blood test results in primary care a qualitative study

**DOI:** 10.3399/BJGP.2022.0069

**Published:** 2022-07-12

**Authors:** Jessica Watson, Chris Salisbury, Penny F Whiting, William T Hamilton, Jonathan Banks

**Affiliations:** Centre for Academic Primary Care;; Centre for Academic Primary Care;; Population Health Sciences, Bristol Medical School, University of Bristol, Bristol.; University of Exeter, Exeter.; Centre for Academic Primary Care, Population Health Sciences, Bristol Medical School, University of Bristol and National Institute for Health Research, Applied Research Collaboration West, Bristol.

**Keywords:** blood tests, diagnostic testing, doctor–patient communication, qualitative research, patient engagement, patient safety

## Abstract

**Background:**

Rates of blood testing in primary care are rising. Communicating blood test results generates significant workload for patients, GPs, and practice staff.

**Aim:**

To explore GPs’ and patients’ experience of systems of blood test communication.

**Design and setting:**

Qualitative interviews with patients and GPs in UK primary care in both urban and rural practices in the West of England.

**Method:**

A total of 28 patients and 19 GPs from six practices were recruited, with a range of socioeconomic and demographic characteristics. Patients were interviewed at two time points: a) at or soon after their blood test and b) after they had received their test results. The GPs who requested the tests were also interviewed (they could complete a maximum of two interviews about different patients). Eighty qualitative interviews were undertaken; 54 patient interviews and 26 GP interviews.

**Results:**

Methods of test result communication varied between doctors and were based on habits, unwritten heuristics, and personal preferences rather than protocols. Doctors expected patients to know how to access their test results. In contrast, patients were often uncertain and used guesswork to decide when and how to access their tests. Patients and doctors generally assumed that the other party would make contact, with potential implications for patient safety. Text messaging and online methods of communication have benefits, but were perceived by some patients as ‘flippant’ or ‘confusing’. Delays and difficulties obtaining and interpreting test results can lead to anxiety and frustration for patients.

**Conclusion:**

Current systems of test result communication are complex and confusing, and mostly based on habits and routines rather than clear protocols. This has important implications for patient-centred care and patient safety.

## INTRODUCTION

Rates of blood testing are rising in primary care.^1^ Blood tests are important for diagnosis and monitoring, but tests in themselves do not make people better, unless actions based on the test result lead to a change in patient management or reassurance. Both are dependent on test result communication. A systematic review of US studies quantifying failures in test result follow-up has shown that between 6.8% and 62% of laboratory tests are not followed-up; no relevant UK research was identified.^2^

Surveys and focus group studies have shown that UK general practices generally rely on patients contacting the practice to obtain their test result, with a lack of fail-safe mechanisms.^3^^–^^5^ Failures to communicate and action abnormal results can lead to delayed and missed diagnoses;^6^ conversely if normal results are not adequately communicated, patients are unlikely to be reassured by testing. This is an important potential source of patient harm, with failure or delay in diagnosis being the commonest cause of malpractice claims in primary care worldwide.^7^ A UK medical protection organisation’s database analysis demonstrated system hazards in management of laboratory tests in 83% of 647 GP practices, with 628 out of 1604 hazards identified being issues relating to communication.^8^ Analysis of 50 UK clinical negligence claims involving test result management systems in general practice found that just under half of cases involved a failure to notify patients of an abnormal test result, and 36% involved a test result not being actioned by a doctor.^9^

Safe and efficient systems of test result communication are particularly important in the current context of rising primary care workload.^10^ The average GP is estimated to spend 1.5 to 2 hours per day reviewing test results;^1^ more efficient systems of test communication could therefore have an impact not only on patient safety but also on GP workload. Recent advances in the use of technology in general practice, such as greater use of text messaging and online patient access to results, have been accelerated during the COVID-19 pandemic and rates of remote consultation have risen.^11^ These technologies offer potential to improve test result communication, but current evidence on the impact of these changes on patient experience is limited.^12^

Studies using focus groups with clinicians and patients have demonstrated variation in systems of test communication between GP practices, lack of fail-safe mechanisms, and frequent delays and dissatisfaction among patients.^3^^,^^13^ In-depth interviews and paired data comparing doctors’ and patients’ experiences of test result communication within a single healthcare encounter have not previously been explored. This study draws on data that examines blood testing, communication, and shared decision making in primary care to explore how test results are communicated to, and accessed, by patients in primary care in the UK.

**Table:** How this fits in

Previous studies have shown that failure to communicate or action blood tests can lead to patient harms, with delay in diagnosis being the commonest cause of malpractice claims in primary care worldwide. This study found that systems of test result communication vary between doctors and are often based on habits, unwritten heuristics, and personal preferences rather than protocols. Doctors generally expect that patients know how to access their test results, and assume that patients will proactively seek out their test results, with implications for patient safety. Practices have an ethical and medicolegal obligation to ensure they have robust systems for test communication.

## METHOD

This study used qualitative interviews with doctors and patients. Interviews were carried out between 31 May 2019 and 17 March 2020. A participating patient’s blood test represented a ‘case’ which was examined by interviewing: a) the patient at the time of testing; b) the patient after the test results had been obtained; and c) the doctor who requested the test. The interviews were part of the first author’s doctoral thesis, which aimed to explore decision making and shared understanding of inflammatory marker blood tests.^14^ These objectives were expanded, based on emerging data, and discussions with the study’s patient and public participation group, who identified systems of test communication in the data and commented on its importance for patients. The objective of this analysis was to explore patients’ understanding and experience of obtaining blood test results and compare this with doctors’ perceptions of test result communication. The study has been reported in keeping with COREQ guidelines.^15^

### Recruitment

All practices in the West of England Primary Care Clinical Research Network were invited to participate by email. Out of 23 expressions of interest, six practices were purposively selected to reflect a range of urban and rural practices, and a range of population characteristics including deprivation, age, and ethnicity. All GPs in participating practices were invited to participate, including locums, salaried GPs, and partners. Out of the six practices recruited, two offered online patient access to test results.

Patients were eligible to participate if they were aged >18 years, had blood tests for inflammatory markers requested by participating GPs, and self-identified as being able to speak English sufficiently for interview. Patients were sampled by sex, age, and socioeconomic status.

Eligible patients were offered study information at the time of testing by their GP or phlebotomist. Interviews were conducted face-to-face at participants’ GP practice at the time of blood testing or soon afterwards at the University of Bristol at the patient’s convenience if preferred. A follow-on telephone interview with the patient was arranged 1–2 weeks later, to explore patients’ experiences of test result communication.

After patient recruitment, the GP who had requested the blood tests was contacted to arrange a telephone interview. Most GP interviews took place after both the first and second patient interview had been completed; all GPs had received the test results at the time of interviewing. Each GP could complete a maximum of two interviews (about different patients), to maximise the range of GPs.

### Interviews

Patient and GP interviews were carried out by the first author, a female practising GP with experience and training in qualitative research methodology. Patients and GPs who were interviewed were informed that the interviewer was a GP; it was emphasised that the interviews were non-judgemental, and were focused on exploring communication around testing, not on scrutinising the clinical decision making.

Interviews were semi-structured, using topic guides based on the research questions but flexible enough to allow exploration of issues raised by the participant; GP interviews lasted on average 19 min (range 9–26 min), initial patient interviews 21 min (9–37 min), with shorter follow-up interviews of 11 min on average (3–21 min). The topic guide (see Supplementary Table S1) was adapted iteratively during the study, using information emerging in early interviews to inform subsequent interviews. GPs had access to the patient’s electronic medical records at the time of interviewing as an aide memoire. Interviews were continued until a diverse sample had been recruited and data saturation achieved across patient and GP interviews, meaning the topic guide was stable with no new codes arising.^16^

### Analysis

Audio recordings were transcribed verbatim by an experienced transcriber. Analysis began when the first transcripts were available, so that data collection and analysis were conducted concurrently. Transcripts were analysed using thematic analysis, involving a mixture of inductive and deductive coding, and constant comparison.^17^ Some of the coding was informed by the research questions and the authors’ pre-existing knowledge and was therefore deductive. However, issues relating to the systems of testing were not part of the original research objectives, so the majority of codes in this analysis were inductive, emerging from patient and GP interviews.

A rigorous and systematic approach to data analysis was adopted that drew on the experience and insight of the wider research team to code and interpret the findings. Two members of the research team independently reviewed four transcripts to develop an initial coding framework. The same coding framework was used for both patient and GP interviews, allowing comparisons within cases (that is between doctors versus patients and before versus after test results) and between cases (that is comparing patients and GPs as a group). This framework was adapted following discussions with the study team and tested on a further three transcripts by two authors. A patient and public contributor panel was also used to check and comment on analysis and data interpretation at an early stage. The first author then took responsibility for ongoing coding and categorisation of the data, using NVivo software for data management.

Categories of data and emerging themes were identified, thematic relationships were identified and written up as descriptive and interpretive accounts.

## RESULTS

In total, 28 patients and 19 GPs from six GP practices were recruited. Eighty interviews were carried out between 31 May 2019 and 17 March 2020; 26 GP interviews and 54 patient interviews (most patients and some GPs were interviewed twice). Tables 1 and 2 summarise the characteristics of participating GPs and patients.

**Table 1.** Characteristics of participating patients (*n* = 28)

**Characteristic**	***N* (%)**
**Sex**	
Female	18 (64)
Male	10 (36)

**Ethnicity**	
White British	23 (82)
Black and minority ethnic	3 (11)
Other non-British	2 (7)

**Age group, years**	
18–24	8 (29)
25–34	3 (11)
35–44	3 (11)
45–54	3 (11)
55–64	3 (11)
65–74	1 (4)
≥75	7 (25)

**Socioeconomic status (based on postcode IMD)**	
1 (most deprived)	2 (7)
2	5 (18)
3	2 (7)
4	4 (14)
5	0 (0)
6	2 (7)
7	2 (7)
8	3 (11)
9	2 (7)
10 (most affluent)	1 (4)
Postcode unavailable	5 (18)

*IMD = Index of Multiple Deprivation.*

**Table 2.** Characteristics of participating GPs (*n* = 19)

**Characteristic**	***N* (%)**
**Sex**	
Female	14 (74)
Male	5 (26)

**Type of GP**	
Partner	13 (68)
Salaried	5 (26)
Locum	1 (5)

**Years’ experience**	
0–4	5 (26)
5–9	2 (11)
10–19	8 (42)
≥20	4 (21)

The proportion of female patients recruited (64%, 18/28) is in keeping with the sex balance of patients receiving inflammatory marker blood tests.^18^ Patients reflected a range of deprivation, age, and ethnicity, and had a range of reasons for testing including symptomatic presentations and chronic disease monitoring. Participating clinicians were 68% GP partners (13/19), 26% salaried GP (5/19) and 74% females (14/19), with a range of years of experience.

Thematic headings are used below to present the findings in relation to communicating test results. Paired quotes are used to show doctors’ and patients’ perceptions of the same clinical encounter where possible. Patient quotes are tagged with their presenting issue.

### Unclear systems of test communication

There were multiple routes available for communication of test results: patients could receive results face to face, by telephone, text message or by letter, and communication could come from a doctor, allied health professional, or from receptionists. Communication of test result could be instigated by the GP practice, or it could be up to the patient to initiate communication. Although practices were purposively sampled that offered online access to blood test results, none of the patients interviewed were aware of this option or had used online access for viewing their test results.

Most doctors made individualised decisions about how to share results depending on their knowledge of the patient, the clinical context, and the test results. Methods of communicating results varied between doctors, even within the same practice, and were based on habits, unwritten heuristics, and personal preferences rather than protocols:

*‘There aren’t protocols that we use. There’s a lot of debate in the practice as to how we manage blood test results in that the onus is always put on the patients to call about the results of the blood tests. So, it depends what the results show. If there’s any significant abnormality that needs action, we normally speak to the patient straightaway.’*

(Doctor 8, Male [M], GP partner, 0–4 years’ experience)

The variation and lack of clear protocols for test communication could be problematic if doctors were away and other clinicians were reviewing and actioning test results on their behalf:

*‘It’s always difficult when people in a practice do different things or if I’m away, so somebody else is filing my results they may do it in a different way. Yeah, I think it’s up to the individual.’*

(Doctor 17, Female [F], GP partner, 10–19 years’ experience)

This variation and lack of clarity about methods of test communication led to uncertainty and confusion for patients. As a result they often used guesswork to decide whether or when to contact the practice for results:

*‘Certain tests can be given to you via text message. If there is, well sometimes its sent to you via paper, letter form, and if there’s any cause for concern sometimes the reception will call you and say can we book you in with, it really does, it differs so much as a change in different methods really for every different type of test, so I just kind of go oh ok, I haven’t heard for a certain amount of time, I’ll call up the reception.’*

(Patient 21, F, aged 25–34 years, abdominal symptoms)

### Assumptions

Doctors often assumed that patients would contact the surgery for their test results, and overestimated how engaged patients would be with their test results. For example, in case 8 the doctor said *‘I know he* [the patient] *would call’*, whereas the patient said *‘I’ve never, ever asked for my test results’*:

*‘His CRP* [C-reactive protein] *is 114, she’s put on there see doctor if still has the symptoms. I think I probably would have contacted him with that CRP result …But I’m sure, I know he would call if he was deteriorating anyway.’*

(Paired quote: Doctor 8, M, GP partner, 0–4 years’ experience)

*‘I’ve never, ever asked for my test results. I’ve always just turned up, had my blood taken, gone away and always with the assumption that if there was anything wrong someone would let me know* [laughs] *.’*

(Paired quote: Patient 8, M, aged 35–44 years, monitoring tests for chronic condition)

Similarly, in the communication between Patient 20 and Doctor 20, the doctor made the assumption that the patient would contact the practice to receive a message that his blood tests needed to be repeated. Although the patient did contact the practice, he felt aggrieved that he had not been informed directly:

*‘I didn’t speak to him, I just put a comment that they were all improving and to repeat in 2 weeks’ time.’*

(Paired quote: Doctor 20, M, GP Partner, 0–4 years’ experience)

*‘So anyway, I waited a week, went there last Friday and asked the lady and she said yeah it was all clear, oh but they wanted you to have a blood test again on one particular thing and I’m thinking well I would have never known that if I hadn’t had come and asked … How many people ring up and ask for their results, how many people make the effort to go down and ask, it’s a bit …Yeah, I thought well it’s a bit lax.’*

(Paired quote: Patient 20, M, aged >75 years, follow-up blood tests after hospital discharge)

These two cases highlight the risks of relying on patient-initiated communication methods, and the potential problems that can arise as a result of the lack of clear systems and lack of fail-safe mechanisms for ensuring test results are communicated. Although doctors generally assumed that patients would contact the practice for their results, they had no way of checking this, as this information was not generally recorded in the medical record:

*‘What wouldn’t be recorded is if the patient rings up and the receptionist tells them.’*

(Doctor 3, M, GP partner, 10–19 years’ experience)

Doctors generally expected or assumed that their patients would know how to access their test results, but patients were often unsure about the best way of doing this, as illustrated by the paired quotes from Doctor 13 and Patient 13 in which the doctor thought that they ‘always’ told the patient to ‘contact us for a result’, but the patient did not have a clear understanding of how to do this:

*‘I always say you must get a result one way or another hearing that it’s either normal or abnormal, you need to make sure you contact us for a result if it hasn’t come through to you.’*

(Paired quote: Doctor 13, F, GP partner, 10–19 years’ experience)

‘ *I don’t know how I do that actually. Maybe I ring up and — probably ring up and just ask the receptionist how I’d go about doing that, ’cos I don’t know if I need like a whole appointment for that, I don’t know if they could send those* [blood test results] *to me. I don’t know how it works.’*

(Paired quote: Patient 13, F, 18–24 years, tiredness symptom)

Although doctors emphasised that patients should ‘always contact us’ for their results, many patients took a fairly passive approach and assumed that if they had not heard anything they could safely assume that everything was normal:

*‘Never presume no news is good news. I always say you need to make sure you contact us for a result if it hasn’t come through to you.’*

(Paired quote: Doctor 13, F, GP partner, 10–19 years’ experience)

*‘I can’t be bothered to ring and wait 20, 30 minutes for an answer, so I just think no news is good news.’*

(Paired quote: Patient 10, F, 55–64 years, chronic disease monitoring)

## Methods of communicating results: phoning reception

The two main methods for test result communication described by patients in this study were phoning to speak to receptionists, or to waiting for a text message.

The system of communicating test results over the phone via receptionists or non-clinical staff was perceived by some patients to be a barrier to accessing test results, as receptionists were unable to provide detailed information about the clinical interpretation of results:

*‘The receptionist said, I told her why I was phoning, I said I haven’t had the results of my blood test and she said yes, oh yes, I’ve looked it up, everything’s fine, no further action. So, I didn’t need to go back to see the doctor and then you think well in a way you’ve got a closure of a sort but not of what you’d originally perhaps come about.’*

(Patient 18, F, aged >75 years, joint symptoms)

Some patients felt unhappy about receiving test results from non-clinical staff members who might not have the training or appropriate expertise that they perceived this task required:

*‘I don’t even bother — I could ring here and find out the results but I don’t know about the receptionists and I’m sure they’re well trained, but do they actually know how to read the blood results?’*

(Patient 10, F, aged 55–64 years)

### Methods of communicating test results: text messages

Although systems of texting patients about their test results was generally perceived positively by doctors, there were mixed views from patients. Some welcomed this as a quick and easy way to get reassurance with normal test results, whereas others felt that text messages did not really convey sufficient information or explanation to allow an understanding of the meaning of these results:

*‘That’s suits me ’cos I know … the doctor explained that if they were normal results, they’d come through text so then I knew when I got the text and I quickly read through I was like oh its fine. But obviously if it was abnormal, I wouldn’t want to receive that by text.’*

(Patient 12, F, aged 25–34 years, neck lump symptom)

Patients perceived text messages were useful for normal test results but ‘obviously’ they wouldn’t want to receive abnormal test results by text message; this contrasted with doctors’ perceptions:

*‘We don’t routinely text normal results. If they were abnormal then I would have either text the patient directly or phone the patient.’*

(Doctor 18, M, GP partner, 0–4 years’ experience)

Text messaging systems were generally designed to prevent two-way communication, making them efficient for doctors. However, when doctors included safety-netting advice asking the patient to get back in touch if they had concerns or questions, patients had no clear route to communicate back to the doctor:

*‘My doctor has said several times in texts if this hasn’t worked let me know if you have any more questions, please get in contact with me, but the only way I know of doing that is by booking an appointment or a phone call … So that’s a confusing communication method for me.’*

(Patient 23, F, aged 18–24 years, gynaecological symptoms)

Some patients even felt that a text was perhaps an inappropriate, or ‘flippant’ way of communicating test results, particularly for those with more complex, ongoing problems:

*‘I’d say in people’s situations like mine where it’s an ongoing thing, I don’t feel that a text message is sufficient … I feel if there’s more investigations to be done from that point on, just sending a blunt text isn’t really sufficient because it means nothing to me … So, I don’t know, it just feels a bit, sometimes when you get a text about something like that it seems a tad flippant.’*

(Patient 21, F, aged 25–34 years, abdominal symptoms)

This contrasted with doctors’ perceptions of patients, most of whom assumed that patients were very happy to receive text message communication:

*‘The patients love them* [text messages] *, generally the feedback’s been very good.’*

(Doctor 27, F, locum, 0–5 years’ experience)

### Methods of communicating test results: online access

Although none of the patients interviewed had used online portals to view their test results, several expressed an interest in having access to their results:

*‘I just wish that I could grab my entire medical results … I mean after all it’s your life you’re looking at. You want to try and look after yourself the best way you can.’*

(Patient 25, M, aged 55–64 years, joint symptoms)

Both doctors and patients perceived that a barrier to this was that test results were not designed in an accessible way for patients, with the risk that this information could cause confusion or anxiety for patients.

### Consequences of unclear systems of test communication

Waiting for blood test results could also lead to anxiety and frustration for patients. A lack of clear systems for communication and uncertainty about how and when they would receive their test results exacerbated this anxiety and frustration and left some patients feeling ‘in limbo’:

*‘So I’ve been in limbo for quite a few days, is he going to ring me today, has he had them back?’*

(Patient 20, M, aged >75 years, follow-up blood tests after hospital discharge)

*‘The fact that I’ve had to chase the results is the annoying thing, ’cos obviously if there’s nothing wrong then there’s nothing wrong, but if there’s something wrong probably need to act on it.’*

(Patient 5, M, aged 35–44 years, chest pain symptoms)

These frustrations were exacerbated by challenges with accessing GP appointments and a lack of continuity, particularly for patients who were told they needed to book a follow-up consultation with a GP, or those who had unanswered questions about their tests:

*‘When they say oh come back and see* [GP] *and you can’t get an appointment or you can’t get that contact, that is frustrating ’cos I think when you’ve seen a specific person for that it is nice for them to explain to you that, you know, what they’re finding as it were.’*

(Patient 8, M, 35–44 years, monitoring bloods for chronic condition)

In contrast, those patients who had a booked follow-up or clear understanding of how to get their test results back found that this knowledge could help reduce uncertainty and:

*‘… take the worry out of the wait.’*

(Patient 6, F, aged 45–54 years, joint symptoms)

## DISCUSSION

### Summary

Methods of communicating test results varied between doctors and were based on habits, unwritten heuristics, and personal preferences rather than protocols. Doctors generally assumed that patients knew how to access their test results, whereas patients were often uncertain and used guesswork to decide when and how to try to access their tests. Patients and doctors often assumed that the other party would make contact, with potential implications for patient safety. Text message and online methods of communication have benefits, but were perceived by some patients as ‘flippant’ and ‘confusing’. Delays and difficulties obtaining and interpreting test results can lead to frustration for patients and are a potential patient safety concern.

### Strengths and limitations

The main strength of this research was the ability to compare doctors’ and patients’ perspectives on the same healthcare encounter, which highlighted mismatches in communication and understanding. The main limitation is that interviews were based on patients’ and doctors’ recollection of the healthcare encounter, rather than direct observation of the doctor–patient interaction. This could lead to recall bias and post hoc rationalisation, particularly for doctors, who might feel defensive when interviewed by a fellow GP. Although it was emphasised that the interviews were non-judgemental, GPs might therefore have overestimated the amount of information they communicated to patients about their blood tests. Most GPs seemed to be comfortable discussing cases with a fellow clinician with shared understanding and were open about sharing uncertainties rather than appearing defensive. Patients did not appear to be influenced by the researcher’s status as a GP and did not query clinical issues or seek alternative clinical views, indicating they recognised the researcher’s role as study interviewer rather than clinician. The benefit of interviewing patients rather than observing consultations is that this method made it possible to identify what patients understand and retain after a consultation. Although two practices that offered online access to blood test results were recruited, none of the patients interviewed had used this, so future research is needed to explore patients’ experiences of reviewing test results online.

All interviews were conducted in the UK in the Bristol, North Somerset and South Gloucestershire region, and were limited to those able to speak English fluently and the findings may not reflect the processes and expectations of testing in other healthcare systems or other cultures.

### Comparison with existing literature

The research is in keeping with a previous survey of UK general practices that demonstrated that most rely on patients contacting the practice for their test results, with a lack of fail-safe mechanisms.^5^ A qualitative study using focus groups with UK clinical and office staff in primary care demonstrated the complexity, lack of standard protocols, and problems with test result communication in primary care,^3^ in keeping with this research. Similarly, a survey of US physicians in primary and secondary care demonstrated that many clinicians lacked methods to ensure test results were received and communicated to patients.^19^

Patients’ perspectives have received relatively little attention; focus group discussions with patients about their preferred methods of test communication highlighted patient dissatisfaction with non-clinical staff relaying results.^4^^,^^13^ This study corroborates these findings and provides new evidence of mismatches between doctor and patient expectations, using paired quotes, which demonstrate the potential safety implications when both doctors and patients assume that the other party is responsible for communicating test results. This is in keeping with evidence from clinical negligence claims,^9^ which show that failure or delay in diagnosis is the commonest cause of malpractice claims in primary care worldwide.^7^

Improving accessibility of blood test results is important as part of a wider move towards patient centredness and shared decision making in medicine.^20^ Evidence suggests that without clear explanation, patients are unlikely to be reassured by normal test results,^21^ potentially leading to additional healthcare visits and further tests.^22^

### Implications for research and practice

These findings highlight the risks of clinicians assuming patients will proactively seek out their test results by making contact with the GP surgery, and the potential problems arising from a lack of clear processes and protocols for test result communication. Good practice consensus statements on laboratory test ordering, handling, and communication in primary care have been produced;^23^ however, evidence on implementation is lacking. Practices and local healthcare systems could employ co-production methods^24^ to improve systems of test communication. This would involve key stakeholders including patients, doctors, and members of the wider healthcare team to develop robust communication systems to ensure patients have access to their test results and are able to understand the implications of their tests, and what the next steps should be. The use of technologies such as text message systems and online access to test results have potential to enhance communication, but if patients’ perspectives are not taken into account these technologies could generate frustration or anxiety. Providing information from the medical records to patients in a way that improves safety and quality of care has been identified by the James Lind Alliance as a top 10 priority area for patient safety.^25^

Practices have a medicolegal and ethical responsibility to ensure they have clear, robust systems for communicating test results; new technologies may be incorporated into these systems but are not a panacea. Failure to ensure safe systems for communicating test results could have significant consequences for patients and practices.
